# GORE VIABAHN VBX Balloon-Expandable Endoprostheses as a Bridging Stent With Branched and Fenestrated Endografts in the Endovascular Treatment of Aortic Aneurysms: Protocol for a Retrospective Multicenter Registry (EMBRACE Study)

**DOI:** 10.2196/78970

**Published:** 2026-02-27

**Authors:** Pietro Dioni, Anders Wanhainen, Jacob Budtz-Lilly, Nuno Dias, Tab Bonny, Luca Bertoglio, EMBRACE study investigator, Luca Bertoglio

**Affiliations:** 1 Division of Vascular Surgery, Department of Clinical and Experimental Sciences (DSCS), University and ASST Spedali Civili Hospital of Brescia, Piazzale Spedali Civili 1, Brescia, 25123, Italy, 39 3395605820; 2 Department of Surgical Sciences, Vascular Surgery, Uppsala University, Uppsala, Sweden; 3 Division of Vascular Surgery, Department of Cardiothoracic and Vascular Surgery, Aarhus University Hospital, Aarhus, Denmark; 4 Vascular Center Malmö, Skåne University Hospital, Malmö, Sweden; 5 W. L. Gore & Associates (United States), Flagstaff, AZ, United States

**Keywords:** aorta, fenestrated, branched, covered, stent

## Abstract

**Background:**

Covered stents commercially available are frequently used off-label in conjunction with fenestrated and branched aortic stent grafts, but there is a lack of dedicated devices.

**Objective:**

This study aims to assess the safety and mid-term clinical performance of a new dedicated covered stent, the GORE VIABAHN VBX Balloon Expandable Endoprosthesis (VBX stent graft), when used as a bridging stent with branched and fenestrated aortic endografts in treating complex abdominal aortic and thoraco-abdominal aneurysms.

**Methods:**

A retrospective, multicenter, single-arm study in the European Union (ClinicalTrials.gov NCT05143138) enrolled patients treated with the VBX stent graft as a bridging stent in branched endovascular repair (BEVAR) and fenestrated endovascular repair (FEVAR) to allow endovascular aneurysm repair between January 2017 and December 2021. Up to 15 sites in Europe were required to enroll a minimum of 220 patients. Patients’ medical records were reviewed by the investigator, and specific data were collected ambispectively for up to 5 years of follow-up from the index procedure. The primary end point is target vessel patency (patient level) through 12 months. The registry was designed to statistically test target vessel patency at 12 months in both FEVAR and BEVAR populations. The hypothesis will be tested separately in the 2 cohorts (fenestrated or branched endovascular repair), using patients with core laboratory imaging results available annually through 5 years. The binomial exact test will be used with a 1-sided 2.5% level of significance to test the null hypothesis.

**Results:**

In total, 259 patients were retrospectively enrolled for a prospective follow-up of 5 years: 136 patients (n=99, 72.8% male; mean age 73, SD 8.9 y) in the BEVAR cohort, 92 patients (n=80, 87.0% male, mean age 72.7, SD 8.1 y) in the FEVAR cohort, and 31 patients (n=17, 54.8% male, mean age 70.9, SD 9.4 y) in the mixed fenestrated or branched endovascular repair cohort. Overall, 662 target vessels were stented with the investigational covered stents: 163 (24.6%) celiac trunk, 192 (29.0%) superior mesenteric artery, and 307 (46.4%) renal arteries. The VBX stent grafts were paired with branches in 451 (68.1%) cases or fenestrations in 211 (31.9%) cases among all Cook Medical stent graft cases. The 1-year results will be published in the fourth quarter of 2025, and the 5-year follow-up results will be analyzed by mid-year 2028.

**Conclusions:**

This study will investigate the VBX stent graft performance in combination with fenestrated and branched aortic grafts to corroborate its use in complex aortic endovascular procedures and support the modification of current device instructions for use.

## Introduction

The renal-mesenteric aortic vessels, which include the celiac trunk, superior and inferior mesenteric arteries, and renal arteries, are variably involved in thoraco-abdominal and complex abdominal aortic aneurysms [1]. Extensive aortic pathologies are currently managed mainly by using fenestrated or branched endovascular repair (F/B-EVAR) with high rates of technical success. It is widely recognized as the technique of choice, offering acceptable mid-term results in terms of mortality and morbidity guidelines [2,3] and supported by international societies [4,5]. In order to both preserve perfusion of visceral vessels and successfully exclude the aortic aneurysm, the fenestrations or branches of the main body of the aortic stent graft are bridged to visceral aortic vessels with covered stents (CSs). However, more than 2 decades after the introduction of the first aortic endoprosthesis, the lack of standardization and limited availability of dedicated ancillary materials for F/BEVAR have left this technique highly dependent on individual physician preferences and choices and the evolution of these ancillary devices [6]. Therefore, different CSs have been used during F/B-EVAR, but none of them were specifically approved for use as bridging stents in combination with the different aortic stent grafts available on the market at the time this study was designed [6]. The performance of different CSs has been investigated in single-center or retrospective registries, and only 1 European CS manufacturer, Bentley (Bentley InnoMed), obtained approval for BeGraft Peripheral in combination with fenestrated endovascular repair (FEVAR; NCT03987035) and is testing BeGraft Peripheral PLUS, in combination with branched endovascular repair (BEVAR; NCT03982940) and Cook Medical aortic stent grafts to modify their instructions for use.

The GORE-sponsored EMBRACE study is being conducted to confirm the clinical performance and safety of the GORE VIABAHN VBX Balloon Expandable Endoprosthesis (VBX stent graft), specifically when used as a bridging stent in combination with F/B-EVAR aortic stent grafts to support and corroborate the change of instruction for use and therefore an on-label use.

## Methods

### Generalities

The EMBRACE study (ClinicalTrials.gov NCT05143138) is a retrospective, multicenter, single-arm registry in the European Union that retrospectively enrolled patients treated with the VBX stent graft as a bridging stent in BEVAR and FEVAR between January 2017 and December 2021, with prospective data collection and follow-up. Patients’ medical records were reviewed by the investigator, and specific data were collected retrospectively and prospectively for up to 5 years of follow-up from the index procedure. Considering consistent device design, documented procedures, available clinical outcomes, and operational approaches, a retrospective and prospective registry was deemed an appropriate observational study to address the research question. Retrospective enrollment was feasible in this case because pre- and postimplant computed tomography scans were routinely performed as part of standard clinical practice. Central imaging analysis of multicenter results was intended to support regulatory approval. To treat complex and thoraco-abdominal aortic aneurysms involving the renal-mesenteric arteries, up to 15 sites (Table 1) in Europe (Denmark, Italy, the Netherlands, Norway, Spain, and Sweden) must enroll a minimum of 220 patients who have received treatment with the VBX stent graft as a bridging stent in F/B-EVAR. Each site cannot contribute more than 10% of the total patient number for each cohort without sponsor approval, with a maximum of 30% of total enrollment for a single site. This observational study was reported in accordance with the STROBE (Strengthening the Reporting of Observational Studies in Epidemiology) statement guidelines.

**Table 1.** Information on the 15 European sites involved in the study.

Institution	Principal investigator	City or country	Enrollment, n (%)
Amsterdam UMC, Vascular Surgery	Arjan Hoksbergen	Amsterdam	32 (12.4)
Hospital Clinico San Carlos	Isaac Martínez	Madrid	27 (10.4)
Policlinico Umberto I	Wassim Mansour	Roma	23 (8.9)
Rigshospitalet, Dep. Vascular Surgery	Timothy Resch	Copenhagen	23 (8.9)
Skane University Hospital, SUS, Malmo	Nuno Dias	Malmo	22 (8.5)
Aarhus University, Department of Thoracic and Cardiovascular Surgery	Jacob Budtz-Lilly	Aarhus	21 (8.1)
Azienda Ospedaliera Complesso Ospedaliero San Giovanni—Addolorata	Rocco Giudice	Roma	21 (8.1)
IRCCS Ospedale San Raffaele, Chirurgia Vascolare	Enrico Rinaldi	Milano	20 (7.7)
IRCCS Ospedale Policlinico San Martino	Giovanni Pratesi	Genova	19 (7.3)
UNN Tromsø	Kare Nordhus	Tromsø	19 (7.3)
Uppsala University Hospital	Anders Wanhainen	Uppsala	16 (6.2)
Haukeland Universitetssjukehus	Antje Butter	Bergen	10 (3.9)
Hospital Clínico Universitario San Cecilio	Luis Miguel Salmerón	Granada	5 (1.9)
St. Olavs Hospital	Frode Manstad Hulaas	Trondheim	1 (0.4)

### Ethical Considerations

**Figure 1.**
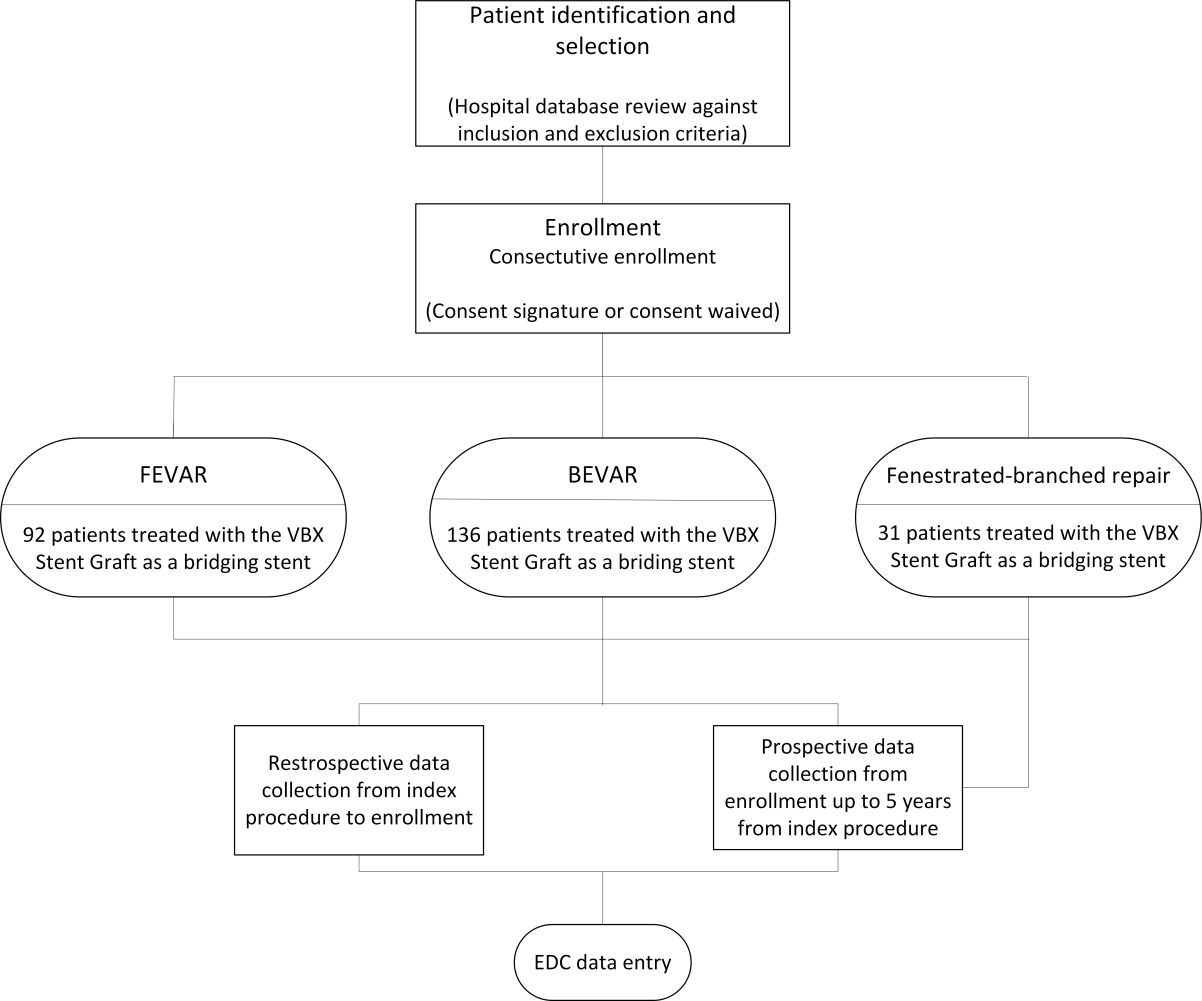
Study design schema. BEVAR: branched endovascular repair; EDC: electronic data capture; FEVAR: fenestrated endovascular repair.

Due to the retrospective nature of this observational study, review by the institutional review board or ethics committee at each of the study sites was ad hoc required in accordance with local regulations and institutional policies regarding minimal-risk observational studies; however, the clinical protocol and all amendments were submitted to the ethics committee for each clinical site [7]. An informed consent form signed by the patient or next of kin or legal representative (for deceased patients at registry entry, unless a waiver was granted), according to local regulations, was required for inclusion in the study. For contacting the potential patient, the investigator must use reasonable methods, including confirming the patient’s survival, looking through medical records, calling readily available numbers, or acquiring contact details from demographic and/or health care registers. All data were deidentified prior to analysis to ensure privacy and confidentiality. The study will be conducted according to the Standards of Good Clinical Practice, the Declaration of Helsinki guidelines, and in compliance with the ISO 14155 standard. No compensation was provided to participants. No identifiable images of participants are included in this study. The study (design illustrated in Figure 1) is registered with ClinicalTrials.gov (NCT05143138).

### Study Population

The inclusion criteria (Textbox 1) reflect the potential broad application of the device as evaluated by the medical judgment of the implanting physician. The inclusion criteria will be assessed during the initial identification, which is part of the retrospective device-use search that considers the patient’s health status at the time of the index procedure. Patients were treated with the VBX stent graft as a bridging stent in conjunction with an F/B-EVAR from December 31, 2021, until January 1, 2017.

Textbox 1. **Inclusion or exclusion criteria.**

**Inclusion criteria**

- Patients treated with the VBX stent graft as a bridging stent in conjunction with a branched or fenestrated stent graft to facilitate endovascular aneurysm repair from December 31, 2021, until January 1, 2017
- Age 18 years or older at the time of implant
- An informed consent form signed by participant or next of kin or legal representative (for deceased patients at registry entry, unless a waiver was granted), according to local regulations

**Exclusion criteria**

- Patient treated for ruptured aneurysm or who were otherwise hemodynamically unstable at the time of the procedure
- Patient treated for acute or subacute dissection, less than 90 days from onset of symptoms
- Patient treated using physician-modified endovascular grafts
- Patient intended to be treated with chimney, periscope, octopus, and sandwich technique per the pre-treatment case plan
- At the time of treatment, patient had known coagulation disorders, including hypercoagulability, that were not amenable to treatment
- Patient was pregnant at the time of treatment
- Participation in another drug or device investigational study within 1 year of device implant, which can confound the registry end points
- Patient had known or suspected systemic infection (including treatment for mycotic aneurysm) at the time of implant

### Study Devices

**Figure 2.**
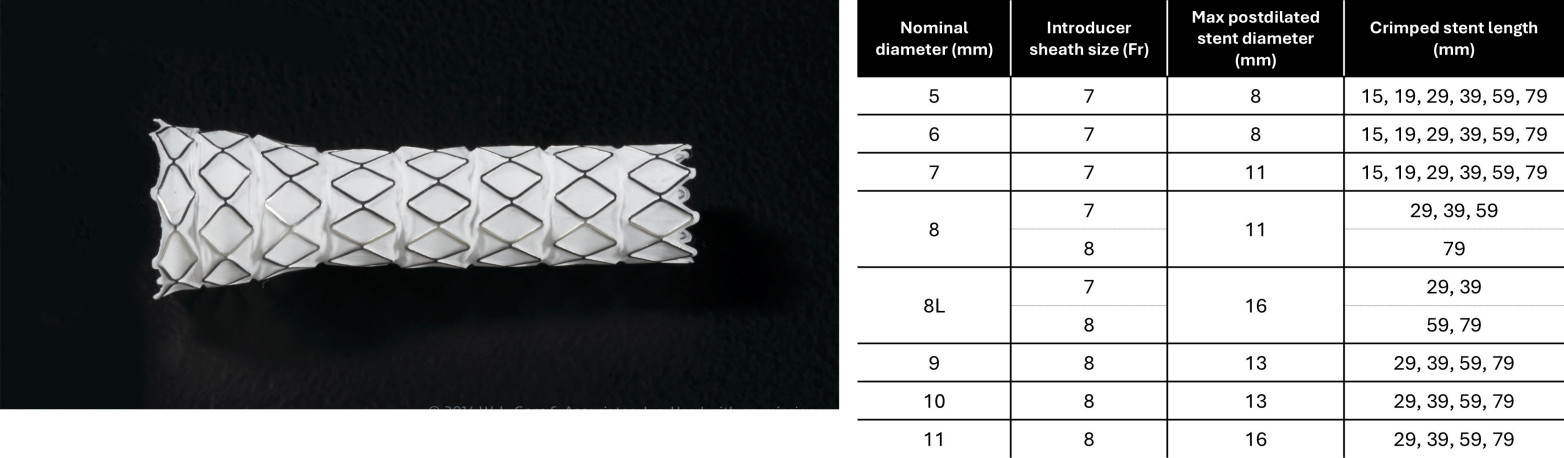
VBX stent graft picture with detailed diameter and length ranges.

The GORE VIABAHN VBX Balloon Expandable Endoprosthesis (hereafter, VBX stent graft; W. L. Gore & Associates, Inc) is made of a balloon-expandable stent graft mounted on a balloon catheter delivery system. The VBX stent graft is made of independent stainless-steel rings that are fully enveloped by expanded polytetrafluoroethylene and fluorinated ethylene propylene film (Figure 2). The delivery system has two radiopaque balloon markers embedded in the shaft to allow accurate delivery of the balloon-expandable stent graft. The delivery system, compatible with 0.035″ (0.89 mm) guide wires, can be used for initial stent placement and subsequent poststent dilatation. The VBX stent graft is available in lengths ranging from 15 to 79 mm and in a variety of diameters, ranging from 5 to 11 mm. The delivery system is also offered in two shaft lengths, 80 and 135 cm. This product is supplied sterile.

### Study Procedure

The sites were expected to perform a search of patients treated with the VBX stent graft as a bridging stent in F/B-EVAR within the hospital database. This search was based on the information already present in the patient’s medical record. Screening was conducted in reverse order, starting with patients treated from December 31, 2021, back to January 1, 2017, or their registry device start date. The research results were recorded in the prescreening list by patients’ index procedure date, arranged from the newest to the oldest patient treated. All patients included in this registry were retrospectively enrolled from participating centers. No prospective enrollment was performed.

### Bias

The registry is meant to retrospectively enroll and prospectively collect data for assessing “real world” clinical experience and patient outcomes during treatment and throughout follow-up up to 5 years. Physicians determine patient selection, diagnostic imaging, and treatment interventions based on this protocol and clinical practice standards. Bias was avoided through strict adherence to the registry protocol, with site compliance monitored, including patient eligibility criteria, per ethics committee regulations. To minimize selection bias, it was mandatory that the enrollment of patients be consecutive in nature based on the date of the index procedure. It is well known that excluding treated patients who are deceased or not reachable at the time of inclusion due to lack of informed consent can generate an important selection bias in a retrospective study and is likely to prevent the achievement of the research objectives. Therefore, for patients who were deceased or not reachable at the time of inclusion, the consent could be waived or signed by next of kin or legal representative, according to local regulations. Additionally, all patients present in the prescreening list who underwent inclusion or exclusion criteria assessment were recorded in the electronic data capture, regardless of enrollment, until the site reached the maximum cap of enrolled patients planned or until the cohort was completed. Potential bias related to the study sponsor’s role in design and oversight will be addressed by strict adherence to the registry protocol. Patient selection, diagnostic imaging, and treatment interventions will be determined by the treating physician based on this protocol and clinical practice standards, independent of the sponsor.

### End Points

The primary and secondary end points are defined in Table 2. Those end points were defined according to the Society of Vascular Surgery reporting standard created for fenestrated and branched endovascular repair [1]. The primary end point is 12-month target vessel patency at the patient’s level. This is defined as uninterrupted patency with no occlusion or procedure performed to maintain patency on the VBX stent graft or native target vessel. Interventions intended to treat endoleak or stent disconnection do not count as loss of primary patency. Patient-level primary patency will be considered lost if any VBX-treated artery loses patency, regardless of the patency status of other treated vessels. End point-related clinical events will be reviewed and adjudicated by the independent clinical events committee (CEC). Secondary and additional end points include all-cause reintervention, VBX-treated artery reinterventions, target vessel technical success, primary technical success, target vessel instability, target vessel patency by both patient-level and vessel-level analysis, aneurysm-related mortality, and major adverse events at 30 days. All study end points will be evaluated annually for up to 5 years following the index procedure. End point analyses will include only arteries treated with a VBX stent graft. Arteries treated during the procedure without a VBX stent graft will be excluded from the analysis.

**Table 2.** Study end point definitions.

Outcome	Definition of SVS^a^ reporting standards
Technical success–related outcomes	
Target vessel technical success	Successful catheterization and VBX stent graft placement in all intended target vessels. For this registry, technical success will be reported for the overall procedure and specifically for the vessels targeted for treatment with the VBX stent graft
Primary technical success (total endovascular procedure)	A modified technical success definition, requiring the following: Successful side branch catheterization and placement of bridging stents with restoration and maintenance of flow in all intended target vessels Patency of all aortic modular stent graft components and intended side branch components Absence of type I or type III endoleaks at completion angiography that extends beyond 30 days by confirmatory imaging (computed tomography angiography, magnetic resonance angiography, or duplex ultrasound)
Vessel patency–related outcomes	
Primary patency (primary end point)	Uninterrupted patency with no occlusion or procedure performed to maintain patency on the VBX stent graft or native target vessel. Interventions intended to treat endoleak or stent disconnection do not count as loss of primary patency
Primary assisted patency	Endovascular intervention performed to maintain patency in the presence of a stenosis before occlusion
Secondary patency	Endovascular restoration of patency after occlusion of the side branch, stent, or stent graft has already occurred. Conversion to bypass or inability to treat by endovascular means defines loss of secondary patency
Occlusion	Objective documentation by angiography, computed tomography, or ultrasound of complete stent occlusion with or without minimal flow into a targeted vessel
Stenosis	Objective documentation by angiography, computed tomography, or ultrasound of stenosis intrastent or into a targeted vessel
Kink	Objective documentation by angiography, computed tomography, or ultrasound of kink in the stented or native segment of a targeted vessel
Other outcomes	
Intraprocedural complications	Any vessel perforation, dissection, or occlusion during target vessel stenting
Reintervention	Any repeated vascular or nonvascular procedure related to the index procedure Reintervention will be adjudicated as major or minor based on the following: Major: deployment of proximal or distal aortic or iliac extensions, removal of the device, use of thrombectomy or thrombolysis, and any major open surgical procedure. Minor: endovascular procedures (percutaneous transluminal angioplasty, atherectomy, stenting) without thrombectomy or thrombolysis, interventions to treat branch vessel stenosis, interventions to treat type II endoleak or branch-related endoleaks, and minor surgical revisions (patch angioplasty) of the access vessel. Each reintervention will be adjudicated as related to a VBX-20–treated branch vessel component, related to a non–VBX–treated branch vessel component, or related to main body components, if possible
Target vessel instability	Death or rupture related to side branch complication (eg, endoleak) or reintervention to treat a branch-related complication, including endoleak, disconnection, kink, stenosis, occlusion, or rupture
Aneurysm-related mortality	Any death that occurs within the first 30 days or any death that results from aneurysm rupture, aorta-related complications (eg, infection, occlusion, dissection, hematoma), or a complication of a secondary intervention
Major adverse events	All-cause mortality Myocardial infarction: Myocardial infarction resulting in severe hemodynamic dysfunction necessitating resuscitation, cardiac arrest, or fatal outcome Respiratory failure requiring prolonged (less than 24 hours from anticipated) mechanical ventilation or reintubation from anticipated) mechanical ventilation or reintubation Any renal function deterioration according to the RIFLE^b^ classification system Bowel ischemia requiring surgical resection or not resolving with medical therapy Permanent paraplegia (any grade 3 A-C spinal cord injury) in a patient who is nonambulatory Any major stroke defined according to National Institutes of Health Stroke Scale or equivalent

a SVG: Society of Vascular Surgery.

b RIFLE: risk, injury, failure, loss of kidney function, and end-stage kidney disease.

### Statistical Method

No randomization or blinding is to be performed due to the confirmatory nature of the study. The registry is designed to statistically test the hypothesis that primary patency of target vessels (primary end point) in F/B-EVAR at 12 months is greater than 77% [8]. The 12-month imaging results will be analyzed, and the hypothesis will be tested separately in the 2 independently powered cohorts (F/B-EVAR). The binomial exact test will be used with a 1-sided 2.5% level of significance to test the null hypothesis (α).

The statistical hypothesis is specified as follows:

- H0: *P*< 77%
- HA: *P*>77%
- α=.025 (1-sided)

However, *P* is the probability of maintaining primary patency at 12 months. The intent-to-treat population will be used to perform this test. A separate analysis of the subgroup of patients treated with mixed endografts (fenestrations and directional branches) as well as other clinical uses not described as only F/B-EVAR will be assessed with no formal hypothesis.

### Sample Size Considerations

This study is designed with the assumptions used for the calculation of sample size, including the anticipated performance of the VBX stent graft in F/B-EVAR, the acceptance criteria determined from the literature, and the derived performance goal. The preliminary clinical evaluation suggested that the acceptable patency of the VBX stent graft used in target vessels at 12 months would be 87%. Given the complexity of the procedure, a noninferiority margin of 10% is considered appropriate. The literature determined that the anticipated 12-month primary patency is assumed to be 89% and 88% in the FEVAR and BEVAR cohorts, respectively [7]. Considering the acceptance criteria of 87% with a noninferiority margin of 10%, the resulting performance goal of 77% for both cohorts, and the expected patency of 89% and 88%, the sample size is determined to be 82 and 98 patients in the F/B-EVAR cohorts, respectively. The calculated sample size in the 2 cohorts will provide 80% power (1-beta) to test the primary hypothesis using a binomial exact test with a 1-sided 2.5% level of significance (alpha). The derived sample size is further inflated by about 18% for the lost-to-follow and missed imaging or visit at 12 months, thereby resulting in 100 and 120 patients to be enrolled in the F/B-EVAR cohorts, respectively.

### Trial Organization and Oversight

An independent CEC will review end point–related clinical events to ensure the consistency of end points reported by the site. The EMBRACE CEC features an interdisciplinary team of 3 members with pertinent expertise who are not directly involved in the conduct of the registry. Sponsors will not participate in any CEC activity and may only tutor the CEC members on the protocol and registry device initially. The core laboratory used is Gore Imaging Sciences. Gore Imaging Sciences will assess migrations, patency, endoleak, and anatomical characterization using objective processes and standard procedures to ensure accuracy, consistency, and objectivity in the results by trained and qualified personnel. Medical imagery will be transmitted to the core laboratory using secure, web portal functionality and will be stripped of personal health information and replaced with numerical patient identifiers in an automated, systemic fashion. Once completed, measurements will be reported directly in the electronic data capture system and considered source data. Study definitions and measurement criteria were standardized prior to image transfer to the core laboratory. The retrospective phase consisted entirely of collecting data from source documents available at each site, covering the period from the time of treatment until the enrollment date, while in the prospective phase, follow-up data were collected prospectively. Diagnostic imaging was performed at the discretion of the physician according to clinical practice standards, and all imaging analyses were conducted exclusively by the core laboratory. Local sites did not participate in the interpretation or adjudication of imaging findings.

## Results

Site enrollment is complete (Table 1), and the data are currently being reviewed by the core laboratory and the CEC. The end point data are anticipated to be available in 2025.

In total, 259 patients were retrospectively enrolled for a prospective follow-up of 5 years. In the BEVAR cohort, 136 patients were enrolled, 99 (72.8%) were male, and the mean age was 73 (SD 8.9) years. In the FEVAR cohort, 92 patients were enrolled, 80 (87.0%) were male, and the mean age was 72.7 (SD 8.1) years. The remaining 31 had both BEVAR and FEVAR procedures (n=17, 54.8% male, mean age 70.9, SD 9.4 y). Of the total patient population, 202 (79.6%) have a history of smoking, 210 (81.0%) present with hypertension, 132 (56.2%) with hypercholesterolemia, and 85 (33.1%) with chronic obstructive pulmonary disease. A total of 662 vessels were treated with a VBX stent graft, including 451 branched and 211 fenestrated vessels. In the BEVAR cohort, 130 (28.8%) of the vessels treated were the superior mesenteric artery, while the remaining vessels were nearly evenly distributed among the celiac trunk (n=116, 25.7%) and left (n=99, 22.0%) and right (n=106, 23.5%) renal arteries. For the FEVAR cohort, the distribution across those vessels was 62 (29.4%), 47 (22.2%), 53 (25.1%), and 49 (23.3%) for the superior mesenteric artery, celiac trunk, and left and right renal arteries, respectively. Of the 14 sites, total enrollment rates ranged from 1 (0.4%) to 32 (12.4%). The range of the enrollment rates for the 3 cohorts was 0 (0%) to 21 (15.4%) for BEVAR, 0 (0%) to 15 (16.3%) for FEVAR, and 0 (0%) to 7 (22.6%) for mixed F/B-EVAR. The 1-year results will be published in the fourth quarter of 2025, and the 5-year follow-up results will be analyzed by mid-year 2028. In the meantime, an intermediate analysis at 3 years will be available in the first and second quarters of 2026, allowing subset analysis of specific populations (ie, connective tissue disorders) or special anatomical analyses (ie, inner vs outer branches)

## Discussion

### Principal Findings

The VBX stent graft is a third-generation stent graft specifically designed for F/BEVAR procedures, and the EMBRACE study is the first controlled study specifically designed to evaluate the outcomes of a covered stent with homogeneous dedicated reporting standards. Not only does it investigate the specific 5-year performance of the VBX stent graft, but also the large real-world patient cohort will provide a dataset available for subset analysis targeted on, for example, the type of vessel, the underlying pathology, and postoperative medications.

The ideal bridging covered stent for use with FEVAR and BEVAR must exhibit a sophisticated equilibrium between high longitudinal flexibility and robust radial strength. This ensures that the device can track through tortuous anatomy and conform to angulated visceral vessels without kinking, while simultaneously resisting compressive forces from the aortic graft or vessel wall. Crucially, the stent design should feature a low-profile delivery system for precise maneuverability and possess predictable flareability at the proximal end, facilitating a secure, seal-tight mating with the fenestration or directional branch to mitigate the risk of type III endoleaks. Finally, the integration of highly radiopaque markers and a biocompatible, thrombosis-resistant encapsulation (typically ePTFE or similar polymers) is essential to ensure accurate deployment and long-term patency. To date, covered stent design and choice have been deeply influenced by the aortic stent graft design. While sealing within reinforced fenestrations is obtained by flaring the proximal edge of the bridging stent, sealing between a branch and its covered stent is based on their overlap. For this reason, stent choice was traditionally guided by the design of the endograft, with balloon-expandable stents (BESGs) preferred for reinforced fenestrations and either self-expanding stents (SESGs) or BESGs for directional branches. Several bridging stents with similar structural features have been used in combination with fenestrated or branched endografts, yielding debated results and pitfalls due to, for example, insufficient radial force to maintain sealing after flaring [6]. The apposition of the bridging stent to the graft at the level of the fenestration is the most frequent site of type III endoleaks in FEVAR [8]. During follow-up, flared stents may become misaligned or rupture at the fenestration, making the selection of the bridging stent and procedural planning critical for long-term freedom from reintervention. To address this issue, several authors have assessed the durability of different bridging stents by simulating aortic physiology in bench tests. These studies demonstrated that the crimped VBX stent graft requires greater force to bend but offers superior flexibility when fully deployed when compared to the Atrium stent (MAQUET Holding B.V. & Co. KG) [9-11]. The data of the FEVAR cohort of the EMBRACE study will be analyzed separately, and postdoc and core laboratory analysis will investigate the conformability and flaring properties of this new stent graft.

Prior research demonstrated the suitability of SESGs in directional branches [12]. However, some endovascular surgeons prefer dedicated BESGs in BEVAR and believe that they may provide clinical value. First, BESGs provide the possibility of performing postdilatation (in situ diameter adjustment) at the distal edge of the stent and inside the branch cuff, which provides flexibility in different anatomical scenarios. Second, the deployment of BESGs is more accurate than SESGs because of the releasing balloon embedded in the stent graft, which allows gradual release and adjustments in case of stent migration. In contrast, SESGs have less precise releasing systems, which may cause displacement from the intended deployment spot, especially in the presence of short sealing zones. Third, trackability remains a topic of debate between the 2 stent graft types. For stents of the same diameter, BESGs are compatible with smaller introducer sheaths compared to SESGs. In cases of narrow endografts and/or aortic lumens, the lower profile of BESGs ensures improved navigability and facilitates catheterization of target vessels [13].

Some limitations associated with BESGs stem from their increased rigidity compared to SESGs; this can lead to kinking of the bridged vessel at the distal edge of the stent. In such cases, a self-expanding bare metal stent is deployed within the BESG to smooth the angle created by the stent graft and restore lumen patency [14]. Stent graft rigidity can also compromise patency in small vessels, especially renal arteries [15,16]. Additionally, renal arteries are more prone to loss of patency compared to splanchnic vessels in branched configurations, regardless of the stent type used for bridging [5,17]. This increased susceptibility may be explained by the kidney’s motion driven by diaphragm movement during breathing [18]. Another limitation of BESG is restricted length availability; for example, the most widely used stent globally, the Atrium (MAQUET Holding B.V. & Co. KG), has a maximum length of 59 mm. This feature may necessitate the use of 2 imbricated BESGs to bridge long distances, thereby increasing the risk of type III endoleak due to component disconnection [19]. In contrast, the VBX stent graft offers lengths ranging from 15 to 79 mm, making it a suitable option for BEVARs that require longer bridging distances.

The VBX stent graft is composed of independent stainless-steel rings that enhance strength and fluoroscopic visibility [20]. Interestingly, independent rings, which are held within a fluoropolymer sleeve that serves as the only linkage for the rings, make the stent less rigid compared to other BESGs, offering an alternative for tortuous vessels. The enhanced flexibility of the stent graft may avoid the use of self-expandable bare metal stents to prevent kinking at the distal sealing zone [21]. As reported in the literature, the performance of the VBX stent graft used as a bridging stent in F/B-EVAR seems reassuring and comparable to other stents available on the market [21-23]. Single-center studies evaluating the performance of the VBX stent graft in F/B-EVAR are essential to better define its indications for use. Previous studies’ results were inconclusive, sparking debate on the application of the VBX stent graft in branched aortic endografts [24,25], where some authors argue that SESGs are preferable [26]. All these considerations justify the effort of the EMBRACE registry to analyze the performance of the VBX stent graft in BEVAR implants with the aim of assessing how its unique features may alter daily practices with a specific focus on renal artery outcomes.

### Study Limitations

The retrospective nature of patient enrollment may introduce selection bias; however, precautions were taken to minimize this risk through standardized inclusion criteria. Variability in the number of patients enrolled in each cohort across sites may help mitigate site-specific biases, enhancing the generalizability of the results, particularly given the inclusion caps. Additionally, due to both the retrospective and confirmatory nature of the study, randomization and blinding were not possible, which may introduce additional potential biases.

### Conclusion

With this study, the authors aim to clarify the role of balloon-expandable covered stents in F/B-EVAR and provide stronger evidence for their use in fenestrated and branched configurations. Once the study end point data are complete, we hope that the findings of this study will refine the indications for endoprostheses in the treatment of abdominal or thoracoabdominal aneurysms, with the inclusion of the VBX stent graft as a dedicated bridging stent in fenestrated or branched aortic endografts.

## List of Abbreviations

BESG: balloon-expandable stent graft
BEVAR: branched endovascular repair
CEC: clinical events committee
CS: covered stent
FEVAR: fenestrated endovascular repair
SESG: self-expanding stent graft
STROBE: Strengthening the Reporting of Observational Studies in Epidemiology
